# Evaluation of *Grewia ferruginea* Hochst ex A. Rich Mucilage as Suspending Agent in Metronidazole Benzoate Suspension

**DOI:** 10.1155/2020/7612126

**Published:** 2020-10-29

**Authors:** Tsadkan Gebremeskel Haile, Gereziher Gebremedhin Sibhat, Ebisa Tadese, Desta Tesfay, Fantahun Molla

**Affiliations:** ^1^Department of Pharmaceutics, School of Pharmacy, College of Health Sciences, Mekelle University, Ethiopia; ^2^Department of Pharmacognosy, School of Pharmacy, College of Health Sciences, Mekelle University, Ethiopia

## Abstract

Various species of the genus *Grewia* have been investigated for different pharmaceutical applications as excipients, yet a study on the potential use of *Grewia ferruginea* mucilage (GFM) as a suspending agent is lacking. Thus, this study is aimed at evaluating the efficacy of *Grewia ferruginea* mucilage (GFM) as a suspending agent in metronidazole benzoate suspension. The suspensions were prepared using 0.5%, 1%, 1.5%, and 2% *w*/*v* of GFM and compared with suspensions prepared from xanthan gum (XGM) and sodium carboxyl methyl cellulose (SCMC) in similar concentrations. The prepared suspensions were evaluated for visual appearance, pH, rheology, sedimentation volume, redispersibility, degree of flocculation, and *in vitro* drug release profile. Stability study was done at different storage conditions for three months. The results indicated that all the prepared suspension formulations exhibited pseudoplastic flow characteristics with viscosity imparting ability of the suspending agents in the order of XGM > GFM > SCMC (*p* < 0.05). The flow rate and redispersibility of the formulations prepared with GFM were significantly lower than those with SCMC and higher than those prepared with XGM. At 0.5% *w*/*v* suspending agent concentrations, the sedimentation volume of the formulations was in the order of XGM > GFM > SCMC (*p* < 0.05). However, at all other concentrations, the sedimentation volume of the formulations prepared with GFM had similar results with XGM but exhibited significantly higher sedimentation volume than SCMC. The formulations with GFM showed a higher degree of flocculation at 0.5% *w*/*v* concentration but were comparable at 1.5% *w*/*v* with XGM containing formulations. The pH, assay, and *in vitro* release profile of all assessed formulations were within the pharmacopial limit. Thus, based on the finding of this study, it can be concluded that *Grewia ferruginea* bark mucilage has the potential to be utilized as a suspending agent in suspension formulations.

## 1. Introduction

Suspensions are dispersed systems where the solid state (internal phase) is dispersed in a liquid/semisolid dispersion medium or an external phase. In the preparation of suspensions, it is important to consider the proper selection and application of additives specifically suspending agents to maintain the stability and accurate dosing of the preparations [1, 2]. Suspending agents could be prepared from synthetic, semisynthetic, and natural origin. One potential natural source with suitable physicochemical properties for use as a suspending agent is *Grewia Ferruginea* Hochst ex A. Rich [3].

*Grewia ferruginea* Hochst ex A. Rich is one of the plant species of the genus *Grewia*, abundantly found in East Africa mainly in Ethiopia, Sudan, Eritrea, and Kenya [4]. It is an indigenous plant to Ethiopia and found in most regions of the country: Tigray, Amhara, Oromia, and South Nations Nationalities and Peoples regions [5–9].

Mucilage and gum fractions from various *Grewia* species have been exploited for their potential application as pharmaceutical excipient in developing different pharmaceutical dosage forms including solid, liquid, and semisolid preparations, and found to exhibit a promising effect as viscosity enhancers, stabilizers, disintegrants, suspending agents, gelling agents, bioadhesives, film coating agents, and binders [10–17].

Although the potential application of several *Grewia* species as suspending agent have been established elsewhere [18, 19], to the best of our knowledge, no report has been established on the potential use of *Grewia ferruginea* mucilage as a suspending agent. Hence, the present study is aimed at evaluating the indigenous plant G*rewia ferruginea* mucilage as an alternative suspending agent in metronidazole benzoate suspension. Metronidazole benzoate is selected as a model drug in this study because it is practically insoluble in water, has a bitter taste, and has poor bioavailability [20]. Hence, on the basis of its physicochemical properties, it is a good candidate to formulate in suspension dosage form.

## 2. Materials and Methods

### 2.1. Materials

Xanthan gum (batch no. 36170328, UNIVAL, China), sodium carboxymethyl cellulose (batch no. 131120, UNIVAL, Germany), propylene glycol (Bulk Medicines, Germany), sorbitol 70% solution (batch no. 02870617), methyl paraben (batch no. 237, ZHEIANG, China), propyl paraben (batch no. 654, ZHEIANG, China), sodium saccharin (batch no. 20151103, UNIVAL, Germany), and metronidazole benzoate (API) (Hubei Hungyanphar, China) were generously donated by Addis Pharmaceutical factory Sh. Co. Tween 80 (LOBA Chemie, India); sucrose (Neolab Life Science Co.), hydrochloric acid 35.4% (LOBA Chemie, India), ammonium acetate 96% (LOBA Chemie, India), methanol 99.8% HPLC grade (LOBA Chemie, India), and injection filters (Co60, novelab) were purchased from local markets. All chemicals used for this experiment were analytical grade and used as received.

### 2.2. Methods

#### 2.2.1. Sample Collection and Mucilage Extraction

Mature, healthy, and fresh stem barks of *Grewia ferruginea* were collected from the farmlands around Enticho district of Tigray region, Ethiopia. The extraction procedure of the mucilage was already published [3]. Briefly, the inner stem bark of *Grewia ferruginea* was peeled off, size reduced, and air-dried for 48 hours. Then, the sample was mixed with distilled water in the ratio of 33.3 g to 1 L, soaked for 48 hours, and filtered using a clean muslin cloth to separate debris. Next, 4.5 L of ethanol (96%) was added to the extract to precipitate it, and the precipitate was further treated with another 4.5 L of ethanol (96%) to completely extract the mucilage. Finally, the mucilage was dried in an Oven (Beschickug 100–800, Germany) at 50°C for 24 hours, powdered to a particle size of 180 *μ*m using a miller (ZAIBA OE-999, Japan), and used for suspension preparations.

#### 2.2.2. Fourier Transform-Infrared (FTIR) Compatibility Study

The compatibility of the mucilage with the drug was checked by preparing a 1 : 1 mixture of *Grewia ferruginea* mucilage and metronidazole benzoate and compressed on an IR press by applying a pressure in a hydraulic press, and the films on the disk were scanned over a wavenumber of 4000-450 cm^−1^ in an IR spectrometer (Shimadzu, IR prestige 21, Japan). The major peaks of IR spectra of metronidazole benzoate in the mixture were analyzed and compared with respect to the IR spectra of metronidazole benzoate alone [21].

#### 2.2.3. Preparation of Suspensions

Suspension formulations containing metronidazole benzoate were prepared with formulas as shown in Table 1. First, the stated amounts of suspending agent and metronidazole benzoate were triturated together with 10 mL of solution containing 15 g sucrose, 0.1 mL Tween 80, and 0.07 g sodium saccharine to form a smooth paste using mortar and pestle. Then, 30 mL sorbitol solution (70%) was gradually added to the prepared paste with constant trituration followed by the addition of preservative solution (methyl paraben and propyl paraben in propylene glycol). The formed mixture was then transferred into a 125 mL amber colored bottle, made up to 100 mL with distilled water, and then shaken vigorously for 5 minutes.

To prepare flocculated suspensions, potassium dihydrogen phosphate with a concentration of 0.54% (*w*/*v*) was used as a flocculating agent. The same step as mentioned above was followed except that the solution of potassium dihydrogen phosphate was added to the smooth paste formed and triturated before the addition of preservative solution [20, 22].

#### 2.2.4. Evaluation of the Suspensions

The suspending ability of *Grewia ferruginea* mucilage, by comparing with sodium carboxymethyl cellulose (SCMC) and xanthan gum (XGM), was evaluated by assessing the stability of the prepared suspensions on the basis of their visual appearance, pH, sedimentation volume, redispersibility, rheology, degree of flocculation, drug content, and dissolution profile.

#### 2.2.5. Appearance and pH of the Suspensions

The bulk appearances of the prepared metronidazole benzoate suspensions were visually examined for color and homogeneity. The pH of each suspension was measured using a pH meter (AD 8000, Japan), which was calibrated prior to use with standard buffer solutions having specified pH.

#### 2.2.6. Viscosity of the Suspensions

To evaluate the effect of suspending agent concentration on viscosity, the viscosity of all the suspension formulations was measured at room temperature with a viscometer (Brookfield RVDVE-8568340 USA) using spindle number 4 at a shear rate of 20 rpm [20]).

The effect of shear rates on viscosity was also studied using a rotational viscometer (Brookfield RVDVE-8568340 USA). Thus, viscosities of the suspensions at a concentration of 0.5% suspending agent were measured in mPas within 48 hours of preparation. The measurements were made at room temperature using spindle number 4 at 20, 30, 50, 60, and 100 rpm [23].

#### 2.2.7. Flowability of the Suspensions

The flow rates of the suspensions were measured based on the method described by Rishabha et al., *[*24*]*. Accordingly, the time required for each 10 mL suspension sample to flow through a 10 mL pipette was used to calculate the flow rate using the Equation (1).

(1) $$\begin{array}{c} \text{Flow rate}=\frac{V_{s}}{T}, \end{array}$$

where *V*_*s*_ is the volume of the sample in the pipette (in mL), and *T* is time (in second) required for the 10 mL suspension to totally elute out of the pipette.

#### 2.2.8. Redispersibility of the Suspensions

A 20 mL sample from each suspension formulations was transferred into a 25 mL measuring cylinder and allowed to settle for one week and one month separately. Redispersibility at 7^th^ day and after a month was evaluated by manually turning the cylinders through a one hundred eighty degree cycle. Redispersibility was recorded as the number of complete cycles required to completely resuspend the formulation in the cylinder [25].

#### 2.2.9. Sedimentation Volume (%)

Twenty milliliters of each suspension formulations were transferred into a 25 mL of graduated measuring cylinder and kept at room temperature. The sedimentation volumes (%) of the formulations were recorded every day for seven days and then the recording continued every week for a total of four weeks. The readings of the sedimentation volumes (%) were taken where the clear supernatant started to become cloudy upon descending from the top surface of the suspension using the Equation (2) [20].

(2) $$\begin{array}{c} \text{Sedimentation volume}\left(\%\right)=\frac{\text{ultimate sediment volume}}{\text{initial volume}}\text{x}100. \end{array}$$

#### 2.2.10. Effect of pH on Sedimentation Volume

To determine the effect of pH, 10 mL suspensions with a pH of 2, 6.5, 8, and 10 were prepared from each of the three suspending agents at a concentration of 0.5% (*w*/*v*). The pH of the suspensions was adjusted using 1 N HCl. pH meter (AD 8000, Japan) was used to check the attainment of the desired pH. Each of the preparations was poured into a 25 mL of graduated measuring cylinder.

The sedimentation volumes (%) of the formulations were noted every day for seven consecutive days after preparation [23].

#### 2.2.11. Degree of Flocculation

The degree of flocculation (*β*) was calculated by comparing the ultimate sedimentation volume of the flocculated system with the ultimate sedimentation volume (F*α*) of the deflocculated suspension as per the Equation (3) [11].

(3) $$\begin{array}{c} \text{Degree of flocculation }\left(\beta \right)=\frac{F}{F\alpha }, \end{array}$$

where *F* is the ultimate flocculation height in the flocculated system, and *Fα* is the ultimate sedimentation height in the deflocculated system.

#### 2.2.12. *In Vitro* Dissolution Study

The *in vitro* dissolution test of the metronidazole tablet [26] was adopted since there is no official specification for the acceptable range of dissolution of metronidazole benzoate suspensions within a specified period of time. The test was conducted in 900 mL of 0.1 N HCl using dissolution apparatus type II (Pharma test, PTWS 820D, Germany) at 100 rpm paddle rotation speed. The formulated suspensions were shaken well for 30 seconds and then a 5 mL suspension was introduced into the center of each vessel by syringe. Samples of 5 mL were withdrawn at 10, 20, 30, 45, and 60 minutes. Each sample withdrawn was replaced with an equal volume of fresh dissolution medium, kept at the same temperature of 37°C, to maintain the sink condition. Each of the sample solutions was filtered using a Whatman No.1 filter paper, appropriately diluted, and analyzed for the drug content at 232 nm using a UV spectrophotometer (UV/VIS spectrometer T80 PG instruments ltd). The cumulative percentages of metronidazole benzoate released from the suspension formulations were calculated based on the established standard calibration curve. Results recorded were averages of six determinations.

#### 2.2.13. Stability Study of the Suspensions

Metronidazole benzoate suspensions were packed in 125 mL amber glass bottles. The packed bottles were placed in a stability chamber (Binder®, England) and maintained at real-time condition of 25°C/60% RH, accelerated condition of 40°C/75% RH, and in a refrigerator (TL4G, Germany) at 4°C for 3 months. In the meantime, samples were collected at 0, 30, 60, and 90 days. Parameters, which could possibly change during storage, such as pH, drug content, and physical appearance were analyzed. The physical appearance of each sample was evaluated by visual inspection (ICH Q1A (R2), 2003).

#### 2.2.14. Assay of Suspensions

The suspensions were assayed for total metronidazole benzoate concentration using the HPLC method as described in BP [27]. A 5 mL sample of each metronidazole benzoate suspension was mixed with 150 mL of methanol; sufficient distilled water was added with mixing to produce 250 mL and centrifuged at 1000 rpm for 10 minutes. One volume was diluted to 10 volumes with methanol (60%). These samples were then analyzed in triplicate using HPLC (Agilent Techno, 1260 Infinity Series Waldborn, Germany).

#### 2.2.15. Statistical Analysis

The data were analyzed statistically using Microsoft Excel and Origin 8 software (Origin Lab Corporation). One way analysis of variance (ANOVA) was applied for comparison of results. Tukey multiple comparison test was used to compare the differences between properties of the different batches of suspensions. At 95% confidence interval, *p* values less than or equal to 0.05 were considered significant.

## 3. Results and Discussion

### 3.1. Fourier Transform-Infrared (FTIR) Compatibility Study

**Figure 1:**
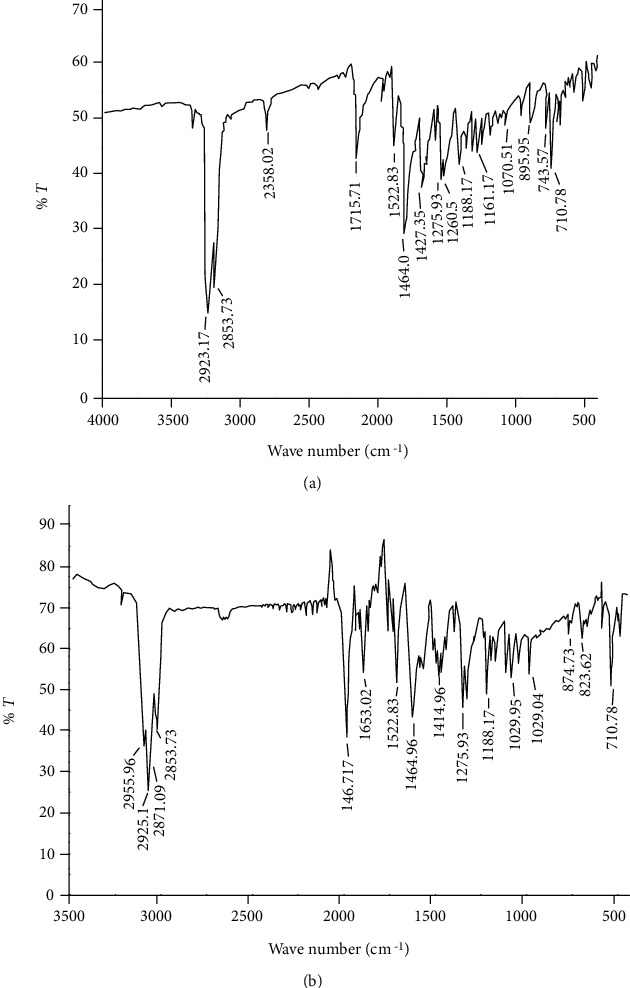
FT-IR spectra of metronidazole benzoate standard (a) and mixture of *Grewia ferruginea* mucilage and metronidazole benzoate (b).

Drug and mucilage interaction was checked by FT-IR spectra. The principal peaks of pure drug (metronidazole benzoate) were 710.78 cm^−1^, 895.95 cm^−1^, 1070.51 cm^−1^, 1188.17 cm^−1^, 1275.93 cm^−1^, 1427.35 cm^−1^, 1464 cm^−1^, 1522.83 cm^−1^, 1715.71 cm^−1^, 2853.73 cm^−1^, and 2923.17 cm^−1^. Pure metronidazole benzoate (MB) exhibited the characteristic bands at 1464 cm^−1^ and 710.78 cm^−1^ that signifies the presence of the aromatic ring attributed to C-C vibrational modes. The ester linkage was distinguished by the strong absorption bands of carbonyl C=O stretching vibrations at 1715.71 cm^−1^. The benzoate ester was also described by the C-O group bands at 1275.93 cm^−1^, 1260.50 cm^−1^, and 1188.17 cm^−1^. The aromatic C-N stretching vibrations were showed by absorption bands at 1427.35 cm^−1^. The bands at 1522.83 cm^−1^ indicated the presence of the nitro group and at 1070.51 cm^−1^ the aliphatic C-N vibrations. These peaks were not affected significantly and remained intact in the physical mixture of MB and GFM as depicted in Figure 1. Thus, it can be suggested that there was no significant interaction between the drug and GFM.

### 3.2. Suspension Evaluations

#### 3.2.1. Physical Appearance of the Suspensions

All suspension formulations prepared from the three suspending agents showed good homogeneity with white-creamy color.

#### 3.2.2. pH of the Suspensions

From Table 2, the pH of all metronidazole benzoate suspensions formulated with GFM, SCMC, and XGM suspending agents are within the range of 5.70 ± 0.07 to 6.421 ± 0.07. This result is within the acceptable pH range of 5.0 to 6.5 for metronidazole benzoate suspensions specified in the BP [27].

#### 3.2.3. Flowability of the Suspensions

Based on the rate and extent of flow out of the pipette, suspensions are classified as less viscous, intermediate viscous, and very viscous. If the suspension totally comes out of the pipette, it is considered as less viscous for which flow rates were calculated. If it comes out partly, it is considered as having an intermediate viscosity. If, however, the suspensions do not totally come out of the pipette, it is considered as viscous [20]. As presented in Table 3, the flow rate decreased with an increase in the concentration of the suspending agent. The flow rates of metronidazole benzoate suspension containing GFM were higher than those containing XGM but lower than those containing SCMC (*p* < 0.05). When equal amounts of suspending agent were used, the flowability of the suspensions was in the order of SCMC > GFM > XGM. This indicates that suspensions prepared from GFM have better pourability from containers than XGM, but less than that of SCMC. The flowability difference among the formulations might be due to the viscosity difference of their respective concentrations. A good quality pharmaceutical suspension is the one that can easily be withdrawn from the container. Therefore, from this result, it can be inferred that GFM has a quality of good suspending agent.

#### 3.2.4. Apparent Viscosity

The apparent viscosity has great importance for stability and pourability or accuracy of dosing for pharmaceutical suspensions. As the apparent viscosity of suspensions increases, fast sedimentation of dispersed particles decreases and makes them to remain suspended for a longer period of time [28].

#### 3.2.5. Effect of Suspending Agent Concentration on Apparent Viscosity

**Figure 2:**
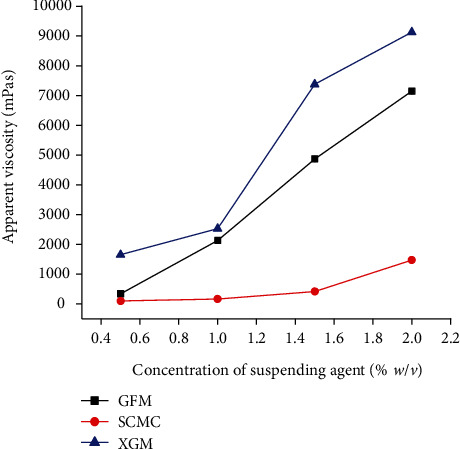
Apparent viscosities of suspensions prepared at different concentrations of suspending agents at a shear rate of 20 rpm (mean ± SD, *n* = 3).

At a fixed shear rate of 20 rpm, the apparent viscosities of all metronidazole benzoate suspensions as a function of different concentrations of suspending agent were measured and the results are presented in Figure 2. Accordingly, as the concentration of the suspending agent increases, the viscosity of the metronidazole benzoate suspensions was increased in all formulations. This suggests that formulations with higher suspending agent concentration are expected to give a suspension that settles slowly. The results of this study also indicate that suspensions containing GFM had exhibited significantly higher viscosity (*p* < 0.05) than those containing SCMC, but showed significantly (*p* < 0.05) lower viscosity than those prepared with XGM. This suggests that metronidazole benzoate suspensions formulated with GFM might have a low terminal settling velocity; thus, the dispersed phase settles at a slower rate and remains dispersed for a longer time; yielding higher stability to the formulated suspension than SCMC but not XGM. Similar results have been reported on the study evaluating *Cassia tora* mucilage as a suspending agent in sulphadimidine suspension [29].

#### 3.2.6. Effect of Shear Rate on Viscosity of the Suspensions

**Figure 3:**
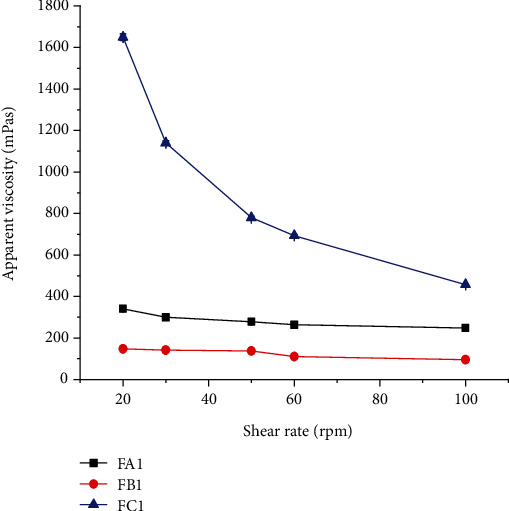
Apparent viscosity of suspension formulations prepared with suspending agents at a concentration of 0.5% *w*/*v* as a function of shear rate (mean ± SD, *n* = 3).

Pseudoplastic flow behavior is a desirable property in suspension formulations in enhancing redispersion and pourability of the suspension prior to administration [30].  Figure 3 presents the viscosity of suspension formulations prepared with suspending agents at a concentration of 0.5% *w*/*v* as a function of shear rate. As the result in Figure 3 shows, the apparent viscosity of all formulations was generally decreased as the speed of rotation increases from 20-100 rpm. This indicated that the suspensions had a pseudoplastic flow property, which might be due to shear-thinning behavior of the suspending agents. Hence, minimum agitation is required for the suspensions to be easily redispersed and a stable dose can be withdrawn.

Therefore, it can be suggested that GFM has the potential to be used as a suspending agent.

#### 3.2.7. Redispersibility of the Prepared Suspensions

Homogeneity at the time of administration of pharmaceutical suspensions highly depends on ease of redispersion of suspensions to have uniform and precise dosing of drugs [30]. The number of inversions required to redisperse the prepared suspensions after a week and a month is presented in Table 4. The number of inversions required to achieve a complete redispersion for SCMC containing formulations was significantly (*p* < 0.05) increased as concentration increases after one week and one month storage time. However, the redispersibility rate of XGM and GFM containing formulations was decreased as concentration increases with the same storage time.

The result of this study also indicates that the XGM containing formulations were more easily redispersed as compared to the GFM containing formulations at 0.5 and 1% *w*/*v* concentration of suspending agent (*p* < 0.05). But at higher concentrations, both XGM and GFM containing formulations did not sediment throughout the four weeks of storage and did not require redispersion, and particles in these formulations were found dispersed throughout the storage time. These results suggest that the increment in viscosity of the XGM and GFM formulations was contributed to retarding the settling velocity of the particles thereby reducing the interparticle interactions and favored ease of redispersibility. The higher redispersibility of SCMC containing formulations might be attributable to the low viscosity and more deflocculated nature of the system. Similar results have been reported in a comparative study of air-dried and freeze-dried *Grewia mollis* gum with SCMC, xanthan gum, and acacia gum as suspending agents in ibuprofen suspensions [11].

#### 3.2.8. Sedimentation Volume (%)

**Figure 4:**
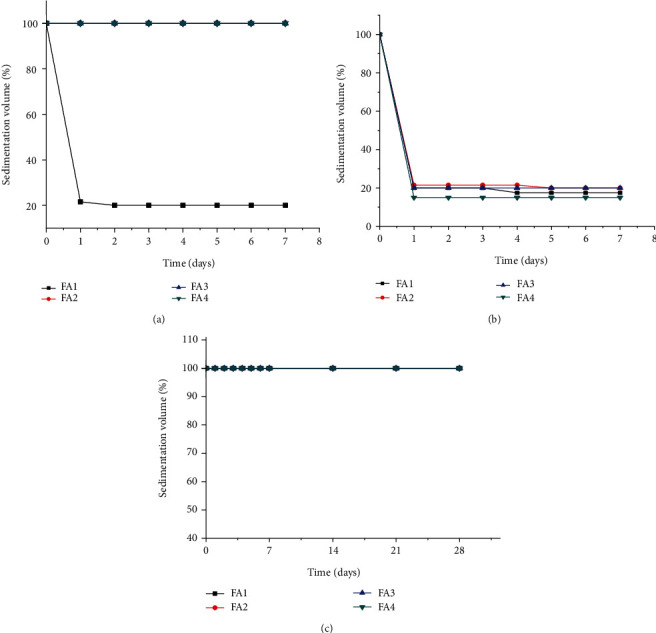
Sedimentation volumes (%) of metronidazole benzoate suspension formulations containing different concentrations of (a) GFM, (b) SCMC, and (c) XGM suspending agents, (*n* = 3, mean ± SD).

Sedimentation volume is the ratio of the height of the sediment after settling to the initial height of the suspension in the cylinder. The larger the ratio, the better is the suspending ability of the suspending agent used [31]. The value of sedimentation volume preferred to have the desired suspension usually accounts to 1 or 100% or approaching to it. Sedimentation volume of near to one or 100% means the particles tend to flocculate easily. The sedimentation volume of the suspensions as a function of storage time is presented in Figure 4.

All XGM containing formulations remained completely suspended (sedimentation volume = 100%) over the 28 days of the study. There was a rapid sedimentation of all SCMC containing formulations after day 1 and a slight decrease to the next consecutive days of storage. At 0.5% *w*/*v*, GFM containing suspensions were rapidly sedimented (17.5% at day one and 15% at day 28), but at other concentrations remained 100% throughout the study time. The ability of the suspending agents to suspend the insoluble drug particles at 0.5% *w*/*v* concentration was in the order of XGM > SCMC > GFM. However, at all other concentrations, GFM exhibited significantly (*p* < 0.05) higher sedimentation volumes than SCMC but comparable results with XGM (*p* > 0.05). High sedimentation volume is an indication that although the internal phase particles have settled, the interparticle attraction and bonding were loose and not strong enough to form a hard cake during the study period. Consistent results have been reported by Ogaji et al., [16] in the study comparing the suspending ability of *Adansonia digitata* gum with sodium carboxymethyl cellulose on paracetamol pediatric suspensions.

#### 3.2.9. Effect of pH on Sedimentation Volume of the Suspensions

**Figure 5:**
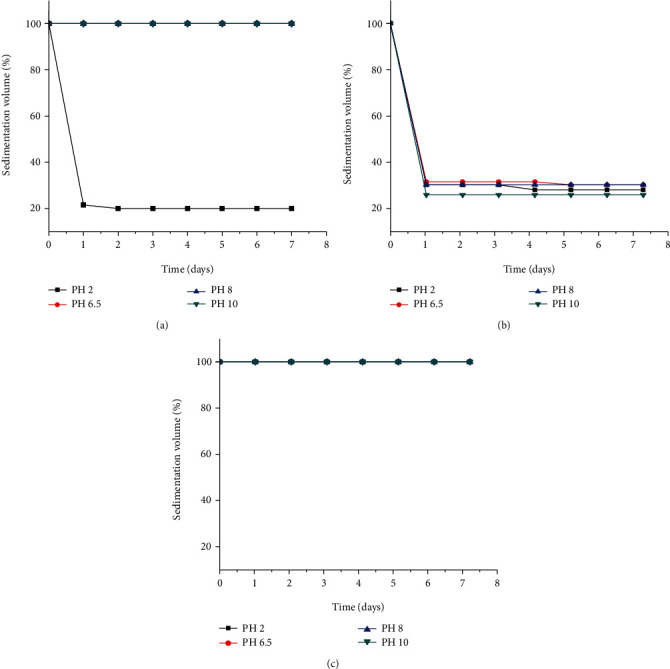
Effect of different pH conditions on the sedimentation volumes (%) of metronidazole benzoate suspensions prepared using 0.5% *w*/*v* of suspending agents ((a) GFM, (b) SCMC, and (c) XGM) (mean ± SD, *n* = 3).

pH change or adjustments using acid or base may affect the surface charge of particles in formulations. The particles may possess positive, neutral, or negative charges which may affect the interaction between the particles and polymer and which in turn may affect the sedimentation profile of these particles. The sedimentation volume of formulations prepared with 1% *w*/*v* suspending agent concentration at different pH values (2, 6.5, 8, and 10) as a function of storage time is presented in Figure 5. For GFM, the sedimentation volume remained 100% throughout the seven days at all pH values except at pH 2, which is 20%. Similarly, all formulations with XGM have 100% sedimentation volume. The results indicated that sedimentation volume remained almost constant with an increase in pH. But the lower sedimentation volume at pH 2 of GFM (20%) might be due to the degradation of the acid-labile peripheral chains of the mucilage at pH 2. This could have led to the lowering of viscosity and also the number of chains that can form a bridge between flocs, which ultimately lowers the (%) sedimentation volume. However, the % sedimentation volume of formulations with SCMC varied in a decreasing fashion at pH 2 (20, 17.5), pH 6.5 (21.5, 20), and almost constant at pH 8 (20) and pH 10 (15) from the first to the seventh day, respectively. Hence, from these results, it can be concluded that GFM is best used as a suspending agent in neutral and alkaline pH values.

#### 3.2.10. Degree of Flocculation (Flocculation/Deflocculation)

**Figure 6:**
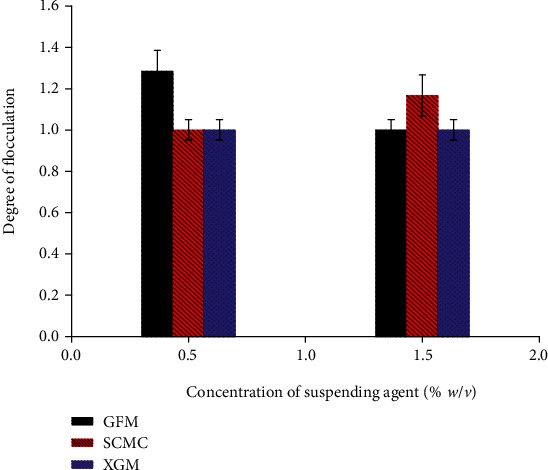
The degree of flocculation of metronidazole benzoate suspension formulations containing 0.5% and 1.5% *w*/*v* suspending agents (mean ± SD, *n* = 3).

Common mechanisms of flocculation in liquid formulations might be due to reduced repulsion between charged particles by polyelectrolytes, by adsorption of nonionic polymers, and free energy changes which result when particles approach each other so closely. This makes the space between the particles too small for polymer molecules in solution [32]. The degree of flocculation of suspensions prepared at 0.5 and 1.5% *w*/*v* suspending agents concentration is depicted in Figure 6. At both concentrations, XAN containing formulations did not sediment but remained highly flocculated throughout the study. At 0.5% *w*/*v* concentration of suspending agent, there was no variation in the degree of flocculation between the formulations of SCMC (1) and XGM (1) but significantly (*p* < 0.05) less flocculated than GFM (1.285). However, at 1.5% *w*/*v* suspending agent, GFM (1) and XGM (1) containing formulations showed the same degree of flocculation and were significantly less flocculated than the SCMC (1.167) containing formulations (*p* < 0.05). This might be due to the presence of electrolytes and their increment with concentration of GFM which might have contributed to the decreasing degree of flocculation.

#### 3.2.11. *In Vitro* Dissolution Profile of Metronidazole Benzoate Suspensions

**Figure 7:**
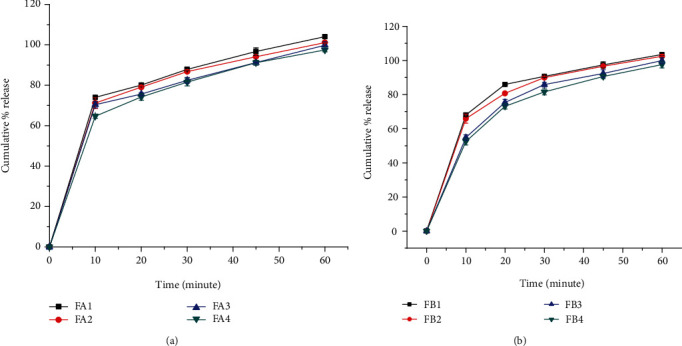
Drug release profiles of metronidazole benzoate suspension formulations containing GFM (a) and SCMC (b) as suspending agent (*n* = 6, mean ± SD).

The dissolution profiles of metronidazole benzoate suspensions formulated with different concentrations of suspending agents are presented in Figures 7(a) and 7(b). The result showed that FB1 (85.91%) released more than 85% of the metronidazole benzoate within 20 minutes whereas FB2 (89.87%), FB3 (85.816%), FA1 (87.8%), and FA2 (86.776%) attained the limit within 30 minutes. FB4 (90.523%), FA3 (91.20%), and FA4 (91.163%) attained the limit within 45 minutes. Therefore, all the formulations prepared with GFM and SCMC as suspending agent released the drug within the USP acceptance range.

The Figures also illustrates that the release rate was faster in formulations containing lower concentrations of suspending agent. This could be attributed to increased viscosity of the formulations with an increase in the concentration of suspending agent. The more viscous the preparation, the slower the release of the API is likely to be. Moreover, the drug release profiles from suspension formulations containing GFM as a suspending agent were slower than those containing SCMC (*p* < 0.05) as a suspending agent at the same concentrations.

#### 3.2.12. Stability Study of the Metronidazole Benzoate Suspensions

Table 5 presents the stability study results of selected batches, FA2 from GFM containing formulations and FB2 from SCMC containing formulations. The suspensions of both formulations were found to have good homogeneity and white creamy in color. Furthermore, both the pH and drug content were within the pharmacopial limits, and the formulations were found to be stable at all storage conditions.

## 5. Conclusion

The results of this study revealed that the suspending properties of *Grewia ferruginea* mucilage in comparison with the reference suspending agents were generally in the order of XGM > GFM > SCMC. The pH, assay, and *in vitro* release profile of metronidazole benzoate in all assessed formulations were within the pharmacopial limit. Thus, based on the present study, it can be concluded that the *Grewia ferruginea* mucilage can be explored as a potential alternative suspending agent in suspension formulations and can be used in low concentration as compared with SCMC.

## Figures and Tables

**Table 1:** Formulation ingredients of the metronidazole benzoate suspensions.

Formula	MB	GFM	SCMC	XGM	MP	PP	T-80	PG	SO	SS	S	DW
	%	%	%	%	%	%	%	%	%	%	%	%
	*w*/*v*	*w*/*v*	*w*/*v*	*w*/*v*	*w*/*v*	*w*/*v*	*v*/*v*	*v*/*v*	*v*/*v*	*w*/*v*	*w*/*v*	*v*/*v*
FA1	4	0.5	—	—	0.18	0.02	0.1	2	30	0.07	15	100
FA2	4	1	—	—	0.18	0.02	0.1	2	30	0.07	15	100
FA3	4	1.5	—	—	0.18	0.02	0.1	2	30	0.07	15	100
FA4	4	2	—	—	0.18	0.02	0.1	2	30	0.07	15	100
FB1	4	—	0.5	—	0.18	0.02	0.1	2	30	0.07	15	100
FB2	4	—	1	—	0.18	0.02	0.1	2	30	0.07	15	100
FB3	4	—	1.5	—	0.18	0.02	0.1	2	30	0.07	15	100
FB4	4	—	2	—	0.18	0.02	0.1	2	30	0.07	15	100
FC1	4	—	—	0.5	0.18	0.02	0.1	2	30	0.07	15	100
FC2	4	—	—	1	0.18	0.02	0.1	2	30	0.07	15	100
FC3	4	—	—	1.5	0.18	0.02	0.1	2	30	0.07	15	100
FC4	4	—	—	2	0.18	0.02	0.1	2	30	0.07	15	100

Key: MB: metronidazole benzoate; MP: methyl paraben; PP: propyl paraben; T-80: Tween 80; PG: propylene glycol; SO: sorbitol solution (70%); SS: sodium saccharine; S: sucrose; DW: distilled water; XGM: xanthan gum; GFM: *Grewia ferruginea* mucilage; and SCMC: sodium carboxymethyl cellulose; FA for GFM, FB for SCMC, and FC for XGM.

**Table 2:** pH of metronidazole benzoate suspension formulations (*n* = 3, mean ± SD).

Concentration of suspending agent (%*w*/*v*)	pH (mean ± SD)		
	GFM	SCMC	XGM
0.5	5.952 ± 0.28	6.311 ± 0.07	6.21 ± 0.014
1	5.790 ± 0.07	6.362 ± 0.14	5.90 ± 0.07
1.5	5.82 ± 0.07	6.365 ± 0.07	5.78 ± 0.07
2	5.721 ± 0.07	6.421 ± 0.07	5.70 ± 0.07

**Table 3:** Flow rate of the suspension formulations at different suspending agent concentrations (*n* = 3, mean ± SD).

Concentration of suspending agent (%*w*/*v*)	Flow rate (mL/sec) (mean ± SD)		
	GFM	SCMC	XGM
0.5	0.175 ± 0.0	0.516 ± 0.01	0.042 ± 0.02
1	0.042 ± 0.01	0.336 ± 0.01	Viscous
1.5	Intermediate	0.184 ± 0.0	Viscous
2	Viscous	0.047 ± 0.0	Viscous

**Table 4:** Average number of turns required to uniformly redisperse sediment of the suspensions after one week and one month.

Suspending agent concentration (%*w*/*v*)	Rate of redispersibility (cycles) (mean ± SD)			
	After a week			After a month		
	GFM	SCMC	XGM	GFM	SCMC	XGM
0.5	16 ± 0.2	15 ± 0.01	13 ± 0.1	17 ± 0.2	16 ± 0.1	14 ± 0.21
1	14 ± 0.1	18 ± 0.1	No	15 ± 0.2	19 ± 0.11	No
1.5	No	20 ± 0.12	No	No	24 ± 0.12	No
2	No	26 ± 0.11	No	No	29 ± 0.13	No

Key: No: no need to redispersed.

**Table 5:** Physicochemical properties of metronidazole benzoate suspension formulations at time zero (Time-0) and after 3 months (Time-3) storage in different storage conditions (*n* = 3, mean ± SD).

Formula	Storage	% of MB (mg/l) (mean ± SD, *n* = 3)		pH	
		Time-0	Time-3	Time-0	Time-3
FA2	25°C/60% RH	100.263 ± 3.964	100.276 ± 0.083	5.79 ± 0.07	5.70 ± 0.06
	40°C/75% RH	101.41 ± 0.05	99.208 ± 0.28	5.79 ± 0.07	5.16 ± 0.07
	4°C	101.42 ± 0.318	100.17 ± 0.055	5.79 ± 0.07	5.8 ± 0.06
FB2	25°C/60% RH	98.52 ± 0.093	98.47 ± 0.264	6.36 ± 0.14	6.5 ± 0.10
	40°C/75% RH	104.2 ± 0.18	100.05 ± 3.59	6.36 ± 0.14	5.9 ± 0.14
	4°C	101.72 ± 0.009	97.74 ± 0.083	6.36 ± 0.14	6.5 ± 0.10

## Data Availability

The data used to support the findings of this study are included within the article. And further data pertaining to the findings of this study are on the hands of the principal investigator. Therefore, requests to access data should be made to [tsadkan.gebremeskel@mu.edu.et].

## References

[1] Kulshreshtha A. K., Singh O. N., Wall G. M. (2010). Pharmaceutical suspensions. From Formulation Development to Manufacturing. *American Association of Pharmaceutical Scientists*.

[2] Swarbrick J., Rubino J. T., Rubino O. P. (2005). *Remington the sciense and practice of pharmacy. Coarse dispersions*.

[3] Haile T. G., Sibhat G. G., Molla F. (2020). Physicochemical characterization ofGrewia ferrugineaHochst. ex A. Rich mucilage for potential use as a pharmaceutical excipient. *BioMed Research international*.

[4] Whitehouse C., Cheek M., Andrews S., Verdcourt B. (2001). Grewia ferruginea A. Rich.(family:TILIACEAE). http://plants.jstor.org/stable/10.5555/al.ap.flora.ftea00536.

[5] Addis G., Asfaw Z., Woldu Z. (2013). Ethnobotany of wild and semi-wild edible plants of Konso ethnic community. *Ethnobotany Research and Applications*.

[6] Addis G., Urga K., Dikasso D. (2005). Ethnobotanical study of edible wild plants in some selected districts of Ethiopia. *Human Ecology*.

[7] Bahru T., Asfaw Z., Demissew S. (2012). Indigenous knowledge on plant species of material culture (construction, traditional arts & handicrafts) used by the Afar & Oromo Nations in & around the Awash National Park Ethiopia. *Global Journal of Human Social Science Geography and Environmental Geosciences*.

[8] Kebede H., Gebrechirstos S. (2016). Floral establishment of major honey plants in Tahtay Qoraro, north western zone of Tigray, Ethiopia. *Bulletin of Environment, Pharmacology and Life Sciences*.

[9] Sileshi A., Gebre-Mariam T., Asres K. (2007). Antibacterial and antifungal activities of extracts of some medicinal plants of Ethiopia. *Ethiopian Pharmaceutical Journal*.

[10] Kumar V. J., Sati O. P., Singh R. (2011). A potential natural tablet binder from *Grewia optiva*. *Scholars Research Library*.

[11] Nep E. I., Conway B. R. (2011). Evaluation of *Grewia* polysaccharide gum as a suspending agent. *International Journal of Pharmacy and Pharmaceutical Sciences*.

[12] Nep E. I., Asare-Addo K., Ghori M. U., Conway B. R. A. M. (2015). Starch-free *Grewia* gum matrices: compaction, swelling, erosion and drug release behavior. *International Journal of Pharmaceutics*.

[13] Nep E. I., Odumosu P. O., Ngwuluka N. C., Olorunfemi P. O., Ochekpe N. A. (2013). Pharmaceutical properties and applications of a natural polymer *fromGrewia mollis*. *Journal of Polymers*.

[14] Ogaji I. J., Hoag S. W. (2011). Effect of *Grewia* gum as a suspending agent on ibuprofen pediatric formulation. *AAPS PharmSciTech*.

[15] Ogaji I. J., Okafor I. S., Hoag S. W. (2013). Grewia gum as a potential aqueous film coating agent I: some physicochemical characteristics of fractions of *Grewia* gum. *Journal of Pharmacy & Bioallied Sciences*.

[16] Ogaji J. I., Omachi J. A., Iranloye T. A. (2012). Effect of *Adansonia digitata* gum on some physicochemical properties of paracetamol pediatric suspension formulations. *International Journal of Research in Pharmacy & Science*.

[17] Shenkute B., Hassen A., Assafa T., Amen N., Ebro A. (2012). Identification and nutritive value of potential fodder trees and shrubs in the mid rift valley of Ethiopia. *Journal of Animal and Plant Sciences*.

[18] Kumar P., Kulkarni G. T. (2012). Characterization of mucilage from *Grewia optiva* as pharmaceutical excipient. *Journal of Chronotherapy and Drug Delivery*.

[19] Sateesha S. B., Balaji S., Rajamma A. J., Shekar H. S., Chandan K. (2013). Prospective of *Grewia* fruit mucilage as gastro retentive drug delivery system: statistical optimization of formulation variables. *RGUHS journal of pharmaceutical sciences*.

[20] Brhane Y., Belete A., Gebre-Mariam T. (2014). Evaluation of local gum of acacia polyacantha as a suspending agent in metronidazole benzoate suspension formulations. *Ethiopian Pharmaceutical Journal*.

[21] Nep E. I., Conway B. R. (2012). Preformulation studies on *Grewia* gum as a formulation excipient. *Journal of Thermal Analysis and Calorimetry*.

[22] Niazi S. K. (2009). *Handbook of pharmaceutical manufacturing formulations*.

[23] Kumbi S. (2013). Carboxymethylation of Dioscorea starch and its evaluation as pharmaceutical suspending agent. *Thesis in Pharmaceutics, School of Graduate Studies*.

[24] Rishabha M., Pranati S., Upendra K., Bhargava C. S., Kumar S. P. (2010). Formulation and comparison of suspending properties of different natural polymers using paracetamol suspension. *International Journal of Drug Development and Research*.

[25] Saeedi M., Dallalpoor-Mohammadi N., Farid D. (2003). Prevention of crystal growth in acetaminophen suspensions by the use of polyvinyl pyrrolidone and bovine serum albumin. *Daru*.

[26] USP XXXII/NF XXVII (2007). *United States Pharmacopoeial Convention*.

[27] BP (2013). *British Pharmacopeia*.

[28] Ayorinde J. O., Odeniyi M. A. (2012). Evaluation of the suspending properties of a new plant gum in Sulphametoxazole formulations. *International Journal of Pharmacology and Pharmaceutical Technology*.

[29] Mann A. S., Jain N. K., Kharya M. D. (2007). Evaluation of the suspending properties of *Cassia tora* mucilage on sulphadimidine suspension. *Asian Journal of Experimental Sciences*.

[30] Lee C. H., Moturi V., Lee Y. (2009). Thixotropic property in pharmaceutical formulations. *Journal of Controlled Release*.

[31] Aremu O. L., Oduyela O. O. (2015). Evaluation of metronidazole suspensions. *African Journal of Pharmacy and Pharmacology*.

[32] Kumar R. S., Yagnesh T. N. (2016). Pharmaceutical suspensions: patient compliance oral dosage forms. *World Journal of Pharmacy and Pharmaceutical Sciences*.

[33] Bhaskar D. A., Uttam K. J., Mahendrasingh A., Jayram C. M., Bhanudas S. R. (2013). Plant exudates and mucilage as pharmaceutical excipient. *Journal of Advanced Pharmacy Education & Research*.

